# α-Fetoprotein mRNA in situ hybridisation is a highly specific marker of hepatocellular carcinoma: a multi-centre study

**DOI:** 10.1038/s41416-021-01363-4

**Published:** 2021-04-06

**Authors:** Shi-Xun Lu, Yu-Hua Huang, Li-Li Liu, Chris Zhiyi Zhang, Xia Yang, Yuan-Zhong Yang, Chun-Kui Shao, Jian-Ming Li, Dan Xie, Xuchen Zhang, Dhanpat Jain, Jing-Ping Yun

**Affiliations:** 1 Department of Pathology, Sun Yat-sen University Cancer Center, State Key Laboratory of Oncology in South China, Collaborative Innovation Center for Cancer Medicine, Guangzhou, China; 2 Key Laboratory of Functional Protein Research of Guangdong Higher Education Institutes and MOE Key Laboratory of Tumor Molecular Biology, Institute of Life and Health Engineering, College of Life Science and Technology, Jinan University, Guangzhou, China; 3 Department of Pathology, The Third Affiliated Hospital, Sun Yat-sen University, Guangzhou, China; 4 Department of Pathology, Sun Yat-sen Memorial Hospital, Sun Yat-sen University, Guangzhou, China; 5 Department of Pathology, Yale University School of Medicine, New Haven, CT USA

**Keywords:** Diagnostic markers, Tumour biomarkers, Hepatocellular carcinoma

## Abstract

**Background:**

Pathologic diagnosis of hepatocellular carcinoma (HCC) can be challenging in differentiating from benign and non-hepatocytic malignancy lesions. The aim of this study was to investigate the potential utility of α-fetoprotein (AFP) mRNA RNAscope, a sensitive and specific method, in the diagnosis of HCC.

**Methods:**

Three independent retrospective cohorts containing 2216 patients with HCC, benign liver lesions, and non-hepatocytic tumours were examined. AFP was detected using ELISA, IHC (Immunohistochemistry), and RNAscope. Glypican3 (GPC3), hepatocyte paraffin-1 (HepPar-1), and arginase-1 (Arg-1) proteins were detected using IHC.

**Results:**

AFP RNAscope improved the HCC detection sensitivity by 24.7–32.7% compared with IHC. In two surgical cohorts, a panel of AFP RNAscope and GPC3 provided the best diagnostic value in differentiating HCC from benign hepatocytic lesions (AUC = 0.905 and 0.811), and a panel including AFP RNAscope, GPC3, HepPar-1, and Arg-1 yielded the best AUC (0.971 and 0.977) when distinguishing HCC from non-hepatocytic malignancies. The results from the liver biopsy cohort were similar, and additional application of AFP RNAscope improved the sensitivity by 18% when distinguishing HCC from benign hepatocytic lesions.

**Conclusions:**

AFP mRNA detected by RNAscope is highly specific for hepatocytic malignancy and may serve as a novel diagnostic biomarker for HCC.

## Background

Hepatocellular carcinoma (HCC) accounts for 70–90% of primary liver cancers worldwide and it remains a leading cause of cancer-related deaths globally.^1,2^ The 5-year survival of patients with HCC remains dismal and this is partially attributed to delayed diagnosis. Guidelines have proposed that the diagnosis of HCC should be based on non-invasive criteria and/or pathology.^3–5^ Although imaging techniques (ultrasound, computed tomography, and magnetic resonance imaging) are routinely used during the diagnosis of HCC, some indeterminate lesions still exist that lack characteristic features upon imaging (enhancement in arterial phase followed by washout in portal venous phase).^6^ Liver biopsies are a critical aspect of the diagnosis in patients with nodules <2 cm and who have cirrhosis.^7^

HCCs represent a spectrum of extremely well-differentiated to poorly differentiated neoplasms, and the diagnosis of this disease poses several challenges for pathologists, particularly in small biopsies. Well-differentiated HCCs must be differentiated from other benign liver tumours, cirrhotic nodules, and sometimes background non-neoplastic parenchyma. On the contrary, poorly differentiated HCCs should be differentiated from intrahepatic cholangiocarcinoma (ICC), metastases, and other primary hepatic neoplasms. Thus, during the pathologic workup of a hepatic mass that is suspected to be HCC, ancillary tests are often required either to confirm the hepatocytic nature of the neoplasm or to support the diagnosis of malignancy in a hepatic lesion depending on the degree of differentiation. Immunohistochemistry (IHC) analyses targeting hepatocyte paraffin-1 (HepPar-1), arginase-1 (Arg-1), polyclonal carcinoembryonic antigen (pCEA), CD10, bile salt export pump (*BSEP*), and glypican-3 (GPC3) have been used to support hepatocytic differentiation,^8^ while IHC analyses of CD34, GPC3, heat shock protein 70 (HSP70), glutamine synthetase (GS), clathrin heavy chain, and enhancer of zeste homologue 2 (EZH2) have been used to support the malignant nature of the hepatocytic neoplasm.^9^ Of these markers, only GPC3 serves both purposes to some extent, although it is not highly specific for hepatocytic differentiation, as it is present in germ cell tumours, melanoma, and other malignancies.^10^ Thus, there is a need for a highly sensitive and specific marker that not only supports hepatocytic differentiation but also aids the identification of malignancy in the evaluation of hepatic mass lesions.

Elevated serum α-fetoprotein (AFP) is a recognised tumour marker for HCC, and its detection by enzyme-linked immunosorbent assay (ELISA) has been used clinically for both screening and diagnosis of HCC.^11^ Although a serum level of AFP > 400 ng/mL is considered to support the diagnosis of HCC, the increase of AFP in individuals with pregnancy, infection, and hepatitis, and other conditions results in confusion when the serum AFP levels are between 20 and 400 ng/mL.^12,13^ In situ detection of AFP using IHC has also been used to demonstrate hepatocellular differentiation as well as to provide supporting evidence of malignancy; however, this method is currently out of favour due to its high background staining and low sensitivity.^14^ The level of AFP mRNA determined by quantitative real-time PCR in peripheral blood has been demonstrated as a promising tumour marker for HCC and can be considered better than serum AFP detection through the use of ELISA.^15,16^ Similarly, detection of AFP mRNA by highly sensitive in situ hybridisation (ISH) technology also provides a significant advance in the detection of AFP protein at a cellular level. The RNAscope method is a significant advancement in technology that addresses the challenges of traditional RNA ISH while providing spatial and morphological resolution at the single-cell level.^17^ This technique employs a unique signal amplification strategy that allows for the visualisation of target RNAs as punctate dots, where each dot represents an individual RNA molecule. The key benefits of the RNAscope assay are high sensitivity due to its signal amplification strategy, high specificity due to the probe design that minimises nonspecific off-target signals, and the ability to detect and quantify of RNA with spatial and morphological context. RNAscope has now become a reliable tool in basic and clinical research for the determination of gene expression in situations where antibody-based detection methods are not very effective.^18–20^

The purpose of this project was to compare the differences and the application value of AFP IHC and RNA in situ detection using a series of liver tumour tissues. We further investigated the diagnostic value of AFP RNAscope, HepPar-1, Arg-1, GPC3, and their combination in distinguishing HCC from benign lesions and non-hepatocytic malignancies in three independent retrospective cohorts from three different centres.

## Methods

### Study population and biospecimen collection

**Fig. 1:**
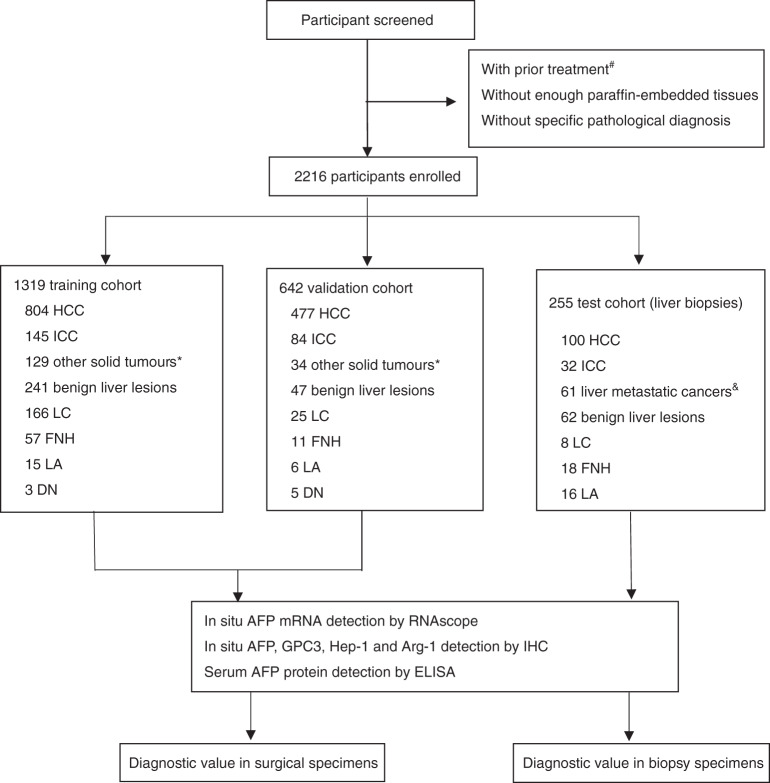
Workflow chart for data generation and analysis. HCC hepatocellular carcinoma, LC liver cirrhosis, LA liver adenoma, DN dysplastic nodule, FNH focal nodular hyperplasia. ^#^Prior treatment, including chemotherapy, radiotherapy, radiofrequency ablation, transcatheter arterial embolisation, and others. *Other solid tumours, including kidney (*n* = 9), urothelial (*n* = 9), cervical (*n* = 8), breast (*n* = 16), lung (*n* = 15), thyroid (*n* = 12), tongue (*n* = 9), laryngeal (*n* = 9), pancreatic (*n* = 9), colorectal (*n* = 18), gastric (*n* = 16), oesophageal (*n* = 13), ovarian (*n* = 11), and endometrial (*n* = 9) carcinoma. ^&^Liver metastatic tumours, including kidney, urothelial, cervical, breast, thyroid, pancreatic, colorectal, gastric, oesophageal, ovarian, endometrial, nasopharyngeal, lung cancer, and neuroendocrine tumours.

Three independent retrospective cohorts (training, validation, and test cohorts) containing 2216 patients with HCC, benign liver diseases, and non-hepatocytic tumours were studied. The flow chart of patients used in this study is presented in Fig. 1. A training cohort comprising 1319 patients from the Sun Yat-sen University Cancer Centre (SYSUCC), Guangzhou, China from January 2015 to September 2017 was recruited. A validation cohort comprising 642 patients diagnosed between March 2015 and October 2017 was also recruited from Sun Yat-sen Memorial Hospital and the Third Affiliated Hospital of Sun Yat-sen University, Guangzhou, China. Another set of liver biopsies (*n* = 255) collected from SYSUCC was used as the test cohort. For all patients, diagnoses were based on clinicopathologic features and histopathologic confirmation. All diagnoses were confirmed by at least two experienced pathologists according to the fifth edition of the World Health Organisation classification of digestive tumours. None of the participants received any prior treatment (chemotherapy, radiotherapy, surgery, or interventional therapy). IHC (AFP, GPC3, HepPar-1, and Arg-1), RNAscope (AFP), and the combination (panel 1: any positive GPC3 IHC and AFP RNAscope; panel 2: any positive GPC3, HepPar-1, Arg-1 IHC, and AFP RNAscope) were used to assess archived formalin-fixed paraffin-embedded (FFPE) samples. The staining was evaluated by three experienced pathologists using blind methods. Two pathologists (S.-X.L. and L.L.L.) provided independent scores for each marker in each case. The scores were entered and evaluated by another investigator (X.Y.). The scores were accepted when the results were consistent. For inconsistent scores, an additional pathologist (J.-P.Y.) provided a third score, and the consistent scores were adopted. Additionally, patient serum was collected (1–14 days before surgery or biopsy) for the detection of serum AFP using ELISA. A serum AFP level > 400 ng/mL was considered to indicate the presence of HCC. This study was approved by the SYSUCC Institute Research Ethics Committee and conducted in accordance with the International Ethical Guidelines for Biomedical Research Involving Human Subjects (CIOMS).

### RNAscope

The RNAscope assay was performed according to the RNAscope^®^ 2.5 BROWN for FFPE manufacturer’s protocol (cat. #322300, Advanced Cell Diagnostics, Hayward, CA, United States). Tissue sections were baked for 1 h at 60 °C, deparaffinised, and treated with pre-treat 1 for 10 min at room temperature. Target retrieval was performed for 15 min at 100 °C, and this was followed by protease treatment for 15 min at 40 °C. Probes were then hybridised for 2 h at 40 °C and then subjected to RNAscope amplification followed by 3,3′-diaminobenzidine (DAB) chromogenic detection. The following RNAscope probes (Advanced Cell Diagnostics, Hayward, CA, United States) were used in this study: dihydrodipicolinate reductase (dapB) (cat. #310043) (negative control), Hs-PPIB (cat. #313908; positive control), and AFP (cat. # 427411).

The stained slides for each sample were analysed using the RNAscope scoring system described in previous studies.^17,21^ RNAscope results were categorised into 5 grades according to the following scoring guidelines: score 0, no staining or <1 dot for every 10 cells (visible at ×40 magnification); score 1, 1–3 dots per cell (visible at ×20–40 magnification); score 2, 4–10 dots per cell with very few dot clusters (visible at ×20–40 magnification); score 3, >10 dots per cell with <10% positive cells having dot clusters (visible at ×20 magnification); score 4, >10 dots per cell with >10% positive cells having dot clusters (visible at ×20 magnification). Samples exhibiting PPIB signal scores of 2 or higher and dapB background scores of 1 or lower were considered to pass the qualification and were included in the analysis presented in this study. Samples possessing PPIB signal scores of ≤2 and dapB background scores of ≥1 were disqualified and omitted from the study. According to the results of receiver operating characteristics (ROC) analyses, cases with RNAscope score ≥ 1 were identified as positive.

### Immunohistochemistry

Tissue microarrays (TMA) consisting of HCC and adjacent non-tumourous liver tissues were constructed using a tissue array instrument (Minicoreexcilone, Minicore, British). For each case, haematoxylin and eosin (H&E)-stained slides were examined, and at least two areas from different regions were marked for sampling. Each tissue core possessing a diameter of 1.0 mm was punched from the marked areas and re-embedded. FFPE HCC sections were de-waxed in xylene and graded alcohols, hydrated, and washed in phosphate-buffered saline (PBS). After pre-treatment in a microwave oven, endogenous peroxidase was blocked with 3% hydrogen peroxide in methanol for 20 min, and this was followed by avidin–biotin blocking using a biotin-blocking kit (K8000, DAKO, Hamburg, Germany). Slides were then incubated with antibodies against AFP (ZA0612, ZSGB-BIO, Beijing, China), HepPar-1 (ZM0131, ZSGB-BIO, Beijing, China), Arg-1 (GT218329, Gene Tech, Shanghai, China), and GPC3 (GT206829, Gene Tech, Shanghai, China) overnight at 4 °C, washed in PBS, and incubated with biotinylated goat anti-rabbit/mouse antibodies (k5007, DAKO, Hamburg, Germany). The slides were developed with DAB and counterstained with haematoxylin.

### Statistical analysis

Statistical analyses were performed using SPSS for Windows (version 19.0). Differences between two independent groups were assessed using Mann–Whitney *U* test (continuous variables and nonparametric analyses). ROC curves were constructed to assess sensitivity, specificity, and respective areas under the curve (AUCs) with 95% confidence intervals (95% CIs). The optimum cut-off value for diagnosis was selected by maximising the sum of the sensitivity and the specificity and minimising the overall error (square root of the sum [1 − sensitivity]^2^ + [1 − specificity]^2^) and also minimising the distance of the cut-off value to the top-left corner of the ROC curve. Net-recognition improvement (NRI) and integrated discrimination improvement (IDI) were performed to compare the diagnostic ability of different markers and panels in HCC diagnosis. *p* Values < 0.05 (two-sided) were considered significant.

## Results

### Expression of AFP mRNA in situ detection by RNAscope

**Fig. 2:**
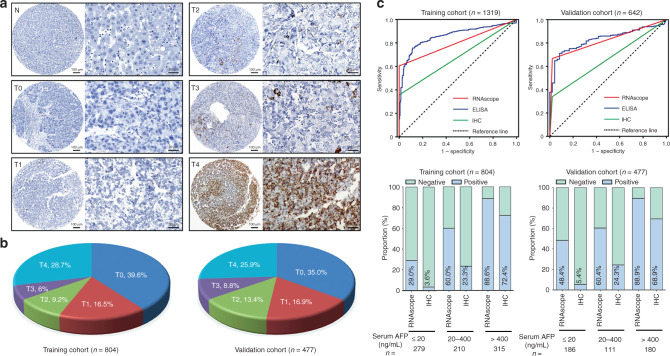
Representative images for AFP RNAscope in paraffin-embedded HCC samples. **a** AFP mRNA was detected by RNAscope in HCC (T) and non-tumourous tissues adjacent to HCC (N). Representative images for scores 0 (T0), 1 (T1), 2 (T2), 3 (T3), and 4 (T4) are shown. **b** Proportion of RNAscope score in HCC tissues in the training and validation cohorts. **c** The ROC curve for AFP detected by RNAscope (AUC = 0.802, *p* < 0.001 and AUC = 0.829, *p* < 0.001), ELISA (AUC = 0.861, *p* < 0.001 and AUC = 0.827, *p* < 0.001), and IHC (AUC = 0.678, *p* < 0.001 and AUC = 0.666, *p* < 0.001) for HCC vs. all other diseases including benign liver diseases and non-hepatocyte tumours in the training and validation cohorts. **d** The positive rates of AFP mRNA and AFP protein in HCC cases with different serum AFP levels are shown in the training and validation cohorts.

In situ expression of AFP mRNA was detected by RNAscope and scored according to the RNAscope scoring system. AFP mRNA was detected primarily in the cytoplasm of cancer cells with variable staining intensity. The scores of 0–4 are shown in Fig. 2a, b. TMA-based RNAscope analysis revealed that AFP mRNA was upregulated in HCC but negative in the adjacent non-neoplastic liver tissue. In the training cohort, the optimal diagnostic cut-off value of AFP RNAscope score for HCC detection vs. other lesions was 0.5, according to the results of ROC analysis (AUC = 0.802, 95% CI 0.779–0.825, sensitivity = 60.4%, specificity = 100%). The same diagnostic potential was confirmed in the validation cohort (AUC = 0.829, 95% CI 0.799–0.860, sensitivity = 66.5%, specificity = 99.4%; Fig. 2c).

### AFP mRNA is a superior marker for detection of AFP than AFP IHC

The diagnostic values of AFP RNAscope and AFP IHC were also studied. Positivity for AFP according to RNAscope was observed in a higher proportion of HCCs compared to AFP IHC (training cohort: 60.4 vs. 35.7%; validation cohort: 66.5 vs. 33.8%; Table 1). All AFP IHC-positive cases were also AFP mRNA positive. Thus, AFP RNAscope is a superior marker for detection of AFP than AFP IHC (AUC = 0.802 vs. 0.678, NRI = 0.248, *p* < 0.001, IDI = 0.196, *p* < 0.001 in the training cohort and AUC = 0.829 vs. 0.666, NRI = 0. 327, *p* < 0.001, IDI = 0.220, *p* < 0.001 in the validation cohort).

**Table 1:** The positive proportion of factors examined in post-surgical samples.

Training cohort	HCC (*n* = 804)	LC (*n* = 166)	FNH (*n* = 57)	LA (*n* = 15)	DN (*n* = 3)	ICC (*n* = 145)	Solid tumours (*n* = 129)
AFP RNAscope	60.4%	0.0%	0.0%	0.0%	0.0%	0.0%	0.0%
AFP IHC	35.7%	0.0%	0.0%	0.0%	0.0%	0.0%	0.0%
AFP ELISA	39.2%	1.8%	1.8%	0.0%	0.0%	0.0%	0.0%
GPC3 IHC	80.0%	15.7%	0.0%	0.0%	33.3%	0.7%	0.8%
HepPar-1 IHC	84.1%	83.1%	96.5%	93.3%	100.0%	2.1%	0.0%
Arg-1 IHC	61.2%	56.6%	61.4%	53.3%	100.0%	0.7%	0.0%

Validation cohort	HCC (*n* = 477)	LC (*n* = 25)	FNH (*n* = 11)	LA (*n* = 6)	DN (*n* = 5)	ICC (*n* = 84)	Solid tumours (*n* = 34)
AFP RNAscope	66.5%	4.0%	0.0%	0.0%	0.0%	0.0%	0.0%
AFP IHC	33.8%	4.0%	0.0%	0.0%	0.0%	0.0%	0.0%
AFP ELISA	37.7%	0.0%	0.0%	0.0%	0.0%	0.0%	0.0%
GPC3 IHC	65.6%	24.0%	0.0%	0.0%	60.0%	2.4%	0.0%
HepPar-1 IHC	72.7%	84.0%	81.8%	66.7%	80.0%	2.4%	0.0%
Arg-1 IHC	69.4%	80.0%	72.7%	83.3%	100.0%	0.0%	0.0%

Test cohort	HCC (*n* = 100)	LC (*n* = 8)	FNH (*n* = 18)	LA (*n* = 16)	DN (*n* = 20)	ICC (*n* = 32)	Metastatic carcinoma (*n* = 61)
AFP RNAscope	45.0%	0.0%	0.0%	0.0%	0.0%	0.0%	0.0%
AFP IHC	28.0%	12.5%	0.0%	0.0%	5%	0.0%	0.0%
AFP ELISA	62.0%	12.5%	0.0%	0.0%	0.0%	0.0%	1.6%
GPC3 IHC	66.0%	0.0%	0.0%	0.0%	20%	3.1%	18.0%
HepPar-1 IHC	81.0%	100%	100%	100%	100%	6.3%	1.6%
Arg-1 IHC	61.0%	100%	100%	100%	100%	0.0%	0.0%

*HCC* hepatocellular carcinoma, *LC* liver cirrhosis, *FNH* focal nodular hyperplasia, *LA* liver adenoma, *DN* dysplastic nodule, *ICC* intrahepatic cholangiocarcinoma, *Solid tumours* 15 types of non-HCC cancers (described in Fig. 1), *Metastatic carcinoma* 14 types of non-HCC cancers (described in Fig. 1), *AFP ELISA* AFP serum level >400 ng/ml was identified positive.

We also studied if the tissue expression of AFP could be detected when serum AFP level was ≤400 ng/mL. As shown in Fig. 2d, Supplementary Fig. 1, and Supplementary Table 1, in the subgroup with serum AFP 20–400 ng/mL, AFP RNAscope was confirmed to be a useful marker for the detection of HCC (AUC = 0.800, sensitivity = 60.0%, and specificity = 100% in the training cohort; AUC = 0.802, sensitivity = 60.4%, and specificity = 100% in the validation cohort). Even in the subgroup with serum AFP ≤20 ng/mL that is considered clinically negative, the AUC was 0.645, the sensitivity was 29.0%, and the specificity was 100% in the training cohort, and the AUC was 0.739, the sensitivity was 48.4%, and the specificity was 99.4% in the validation cohort. This indicated that, regardless of the serum level of AFP, HCC cases can be identified by AFP mRNA as assessed by RNAscope.

### Diagnostic performance of AFP mRNA in situ detection in HCC compared to other IHC markers in surgical specimens (training and validation cohorts)

**Fig. 3:**
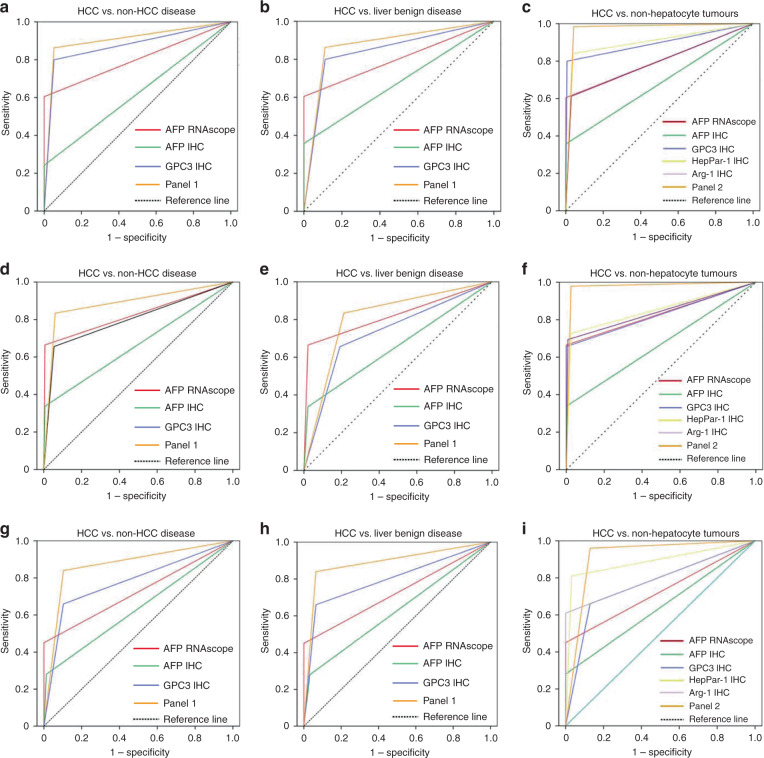
Diagnostic performance of AFP mRNA for in situ detection in the diagnosis of HCC. ROC curve analyses of AFP RNAscope, GPC3, HepPar-1, and Arg-1 and also for panel 1 (any positive of AFP RNAscope +GPC3 IHC) and panel 2 (any positive of AFP RNAscope, GPC3, HepPar-1 and Arg-1) in the diagnosis of HCC. We assessed HCC vs. non-HCC diseases, liver benign diseases, and non-hepatocytic tumours in the training cohort (**a**–**c**), validation cohort (**d**–**f**), and test cohort (**g**–**i**).

AFP and GPC3 are the two markers that not only support hepatocytic differentiation but also support the malignant nature of hepatic mass lesions. Based on our finding that the sensitivity and specificity of AFP RNAscope were both superior than AFP IHC, we next compared the diagnostic efficacy of AFP RNAscope and GPC3 in the differentiation of HCC from non-HCC diseases. As shown in Tables 1 and 2 and Fig. 3a, d, although it possessed a lower sensitivity, AFP RNAscope (AUC = 0.802 in the training cohort and AUC = 0.829 in the validation cohort) exhibited a comparable diagnostic value to that of GPC3 (AUC = 0.872 in the training cohort and AUC = 0.795 in the validation cohort). With a NRI = −0.141 (*p* < 0.001) and IDI = −0.155 (*p* < 0.001) in the training cohort and NRI = 0.069 (*p* = 0.044) and IDI = 0.066 (*p* = 0.069) in the validation cohort, a combination of AFP RNAscope and GPC3 (AUC = 0.903 In the training cohort and AUC = 0.881 in the validation cohort) yielded improved performance in HCC detection compared to GPC3 alone (NRI = 0.063, *p* < 0.001 and IDI = 0.099, *p* < 0.001 in the training cohort, and NRI = 0.172, *p* < 0.001 and IDI = 0.215, *p* < 0.001 in the validation cohort).

**Table 2:** Results for measurement of different markers and panels in the diagnosis of HCC in training, validation and test cohort.

	Training cohort			Validation cohort			Test cohort		
	AUC (95% CI)	Sensitivity	Specificity	AUC (95% CI)	Sensitivity	Specificity	AUC (95% CI)	Sensitivity	Specificity
*HCC vs. non-HCC diseases*									
AFP RNAscope	0.802 (0.779–0.825)	0.604	1.000	0.829 (0.799–0.860)	0.665	0.994	0.725 (0.656–0.794)	0.450	1.000
AFP IHC	0.678 (0.650–0.707)	0.357	1.000	0.666 (0.624–0.708)	0.338	0.994	0.634 (0.560–0.707)	0.280	0.987
GPC3 IHC	0.872 (0.852–0.892)	0.800	0.944	0.795 (0.759–0.831)	0.656	0.933	0.778 (0.716–0.841)	0.660	0.897
Panel 1	0.903 (0.885–0.922)	0.863	0.944	0.881 (0.851–0.911)	0.834	0.963	0.868 (0.818–0.919)	0.840	0.897
*HCC vs. liver benign diseases*									
AFP RNAscope	0.802 (0.777–0.827)	0.604	1.000	0.822 (0.778–0.865)	0.665	0.979	0.725 (0.649–0.801)	0.450	1.000
AFP IHC	0.678 (0.647–0.710)	0.357	1.000	0.658 (0.593–0.723)	0.338	0.979	0.624 (0.539–0.709)	0.280	0.968
GPC3 IHC	0.851 (0.825–0.877)	0.800	0.901	0.732 (0.662–0.803)	0.656	0.809	0.798 (0.729–0.867)	0.660	0.935
Panel 1	0.905 (0.613–0.923)	0.863	0.961	0.811 (0.740–0.881)	0.834	0.787	0.888 (0.832–0.943)	0.840	0.935
*HCC vs. non-hepatocyte tumours*									
AFP RNAscope	0.803 (0.778–0.827)	0.604	1.000	0.832 (0.801–0.863)	0.665	1.000	0.725 (0.653–0.797)	0.450	1.000
AFP IHC	0.679 (0.611–0.746)	0.357	1.000	0.669 (0.623–0.714)	0.338	1.000	0.640 (0.562–0.718)	0.280	1.000
GPC3 IHC	0.897 (0.879–0.915)	0.800	0.993	0.828 (0.796–0.860)	0.656	1.000	0.765 (0.697–0.834)	0.660	0.871
HepPar-1 IHC	0.901 (0.882–0.921)	0.841	0.961	0.851 (0.820–0.883)	0.727	0.975	0.889 (0.838–0.940)	0.810	0.968
Arg-1 IHC	0.791 (0.765–0.817)	0.612	0.971	0.843 (0.812–0.874)	0.694	0.992	0.805 (0.741–0.869)	0.610	1.000
Panel 2	0.971 (0.957–0.985)	0.984	0.958	0.977 (0.960–0.995)	0.979	0.975	0.915 (0.870–0.961)	0.960	0.871

*AUC* area under curve, *95% CI* 95% confident interval, *AFP* alpha-fetoprotein, *IHC* immunohistochemistry.

The pathologic diagnosis of HCC can be challenging sometimes in differentiating from benign lesions and non-hepatocytic malignancies. It is very difficult to distinguish well-differentiated HCC from hepatocellular adenoma and dysplastic nodule (DN) using H&E staining alone. Our results suggested that the combined detection of AFP mRNA and GPC3 could be a promising diagnostic panel to differentiate HCC from other benign liver lesions. ROC curve analyses revealed that the combination of AFP mRNA and GPC3 improved diagnostic performance for HCC compared to that of GPC3 alone (Fig. 3b, e and Table 2; NRI = 0.063, *p* < 0.001 and IDI = 0.103, *p* < 0.001 in the training cohort and NRI = 0.155, *p* < 0.001 and IDI = 0.106, *p* < 0.001 in the validation cohort). Additionally, the specificity of AFP mRNA in HCC was significantly higher than that of GPC3. GPC3 expression was observed in the livers with liver cirrhosis (LC; 16.8%, 32/191) and DN (50.0%, 4/8). Both AFP mRNA and AFP IHC were positive in one LC case in the validation cohort. All three markers were consistently negative in all focal nodular hyperplasia (FNH) and liver adenoma (LA) cases.

Poorly differentiated tumours generally lack morphological features of HCC, and various markers have been suggested to confirm hepatocytic differentiation. In this study, we examined the expression of AFP mRNA, GPC3, HepPar-1, Arg-1, and AFP protein in HCC, ICC, and other solid tumours. As shown in Table 1, AFP mRNA was not expressed in non-hepatocytic malignancies (Supplementary Fig. 2), and the specificity was higher than that of GPC3, HepPar-1, and Arg-1. HepPar-1 (5/229) and Arg-1 (1/229) were positive in a small number of cases of ICC. GPC3 was positive in a small number of non-hepatocytic solid tumours (ICC [3/229] and one oesophageal squamous cell carcinoma [1/13]). AFP RNAscope was positive in cases that GPC3, HepPar-1, or Arg-1 were negative (GPC3-negative cases: 31.7% [51/161] and 51.8% [85/164]; HepPar-1-negative cases: 75.0% [96/128] and 64.4% [84/130]; Arg-1-negative cases: 66.0% [206/312], 71.2% [104/146], respectively, in the training and validation cohorts). ROC curve analyses suggested that the combined panel of AFP mRNA+GPC3+HepPar-1+Arg-1 IHC provided the best results for distinguishing HCC from non-hepatocyte tumours, and the diagnostic value was significantly higher than that for these markers alone (Fig. 3c, f and Table 2). The results indicated that a combined panel of AFP mRNA and GPC3, HepPar-1, and Arg-1 improved the diagnostic performance in diagnosing HCC.

### Diagnostic performance of AFP mRNA in situ detection in HCC compared to other IHC markers in liver biopsies (test cohort)

The diagnostic implication of AFP RNAscope was further evaluated using 255 liver biopsies (test cohort). As shown in Table 3, the positive rates for AFP mRNA, AFP, GPC3, HepPar-1, and Arg-1 in HCC cases (*n* = 100) were 45, 28, 66, 81, and 61%, respectively. AFP RNAscope was only expressed in HCC and possessed a positive predictive value (PPV) of 100% in distinguishing HCC from benign lesions and non-hepatocytic malignancies (Table 1). AFP RNAscope further diagnosed 18/34 HCC cases that were GPC3 negative and 5/13 of HCC cases that were both HepPar-1 and Arg-1 negative.

**Table 3:** Degree of diagnostic accuracy in HCC vs. non-hepatocytic malignancy (NHM) nodules and non-malignant (NM) nodules in liver biopsy (test cohort).

HCC vs. NM	HCC (*n* = 100)	NM (*n* = 62)	Sensitivity	Specificity	PPV	NPV	Accuracy
Both positive	26	0	26.0%	100%	100%	45.6%	54.3%
At least one positive	84	4	84.0%	93.5%	95.5%	78.4%	87.7%
AFP RNAscope	45	0	45.0%	100%	100%	53.0%	66.0%
GPC3 IHC	66	4	66.0%	93.5%	94.3%	63.0%	76.5%

HCC vs. NHM	HCC (*n* = 100)	NHM (*n* = 93)	Sensitivity	Specificity	PPV	NPV	Accuracy
All positive	18	0	18.0%	100%	100%	53.1%	57.5%
At least one positive	96	12	96.0%	87.1%	88.9%	95.3%	91.7%
AFP RNAscope	45	0	45.0%	100%	100%	62.8%	71.5%
GPC3 IHC	66	12	66.0%	87.1%	84.6%	70.4%	76.2%
HepPar-1	81	2	81.0%	97.8%	97.6%	82.7%	89.1%
Arg-1	61	1	61.0%	98.4%	98.4%	70.2%	79.3%

*HCC* hepatocellular carcinoma, *PPV* positive predictive value, *NNP* negative predictive value.

We next examined whether the tissue expression of AFP could be detected at a serum AFP ≤400 ng/mL in liver biopsies. As shown in Supplementary Fig. 1G–I and Supplementary Table 1, in the subgroup with serum AFP 20–400 ng/mL, the sensitivity of RNAscope was 23.5%. Even in the subgroup with serum AFP ≤20 ng/mL, the sensitivity of RNAscope was 14.3%. The data indicated that, regardless of the serum level of AFP, HCC cases could be detected according to AFP mRNA levels assessed by RNAscope.

The diagnostic efficacy of AFP RNAscope and GPC3 in the differentiation of HCC and non-HCC disease in liver biopsies was investigated. As shown in Fig. 3g and Table 2, AFP RNAscope (AUC = 0.725) possessed a comparable diagnostic value to that of GPC3 (AUC = 0.778, NRI = −0.107, *p* = 0.252 and IDI = 0.011, *p* = 0.889). A combination of AFP RNAscope and GPC3 (AUC = 0.868) provided improved performance in HCC detection compared GPC3 alone (NRI = 0.180, *p* < 0.001 and IDI = 0.204, *p* < 0.001).

The application of AFP RNAscope to differentiate HCC from benign hepatocytic lesions and non-hepatocytic malignancies was further explored. As shown in Fig. 3h and Table 2, a panel of AFP RNAscope and GPC3 yielded the best AUC (0.888) in differentiating benign lesions from HCC. When at least one of these two markers were positive, the sensitivity, specificity, PPV, negative predictive value (NPV), and accuracy values for differentiating HCC from non-malignant nodules were 84.0, 93.5, 95.5, 78.4, and 87.7%, respectively (Table 3). The panel including AFP RNAscope, GPC3, HepPar-1, and Arg-1 provided the best AUC (0.915) for the differentiation of HCC from non-hepatocytic malignancies. When at least one of these two markers were positive, the sensitivity, specificity, PPV, NPV, and accuracy values for differentiating HCC from non-malignant nodules were 96.0, 87.1, 88.9, 95.3, and 91.7%, respectively (Table 3).

## Discussion

AFP IHC is sometimes used as a marker in pathologic diagnosis of HCC. The purpose of this study was to compare the difference and diagnostic value of AFP IHC and RNA in situ detection using a series of liver tumour tissues. Our results revealed that the RNAscope detection of AFP is superior than IHC and ELISA for pathological diagnosis of HCC. AFP RNAscope was approximately 24.7–32.7% more accurate in diagnosis of HCC than IHC. Also, nearly all the cases that were positive for AFP protein detected by IHC were also positive for AFP RNAscope. Furthermore, AFP RNAscope could diagnose HCC at different serum levels, even in cases with serum AFP levels ≤20 ng/mL.

The diagnosis of liver lesions can be challenging for pathologists, particularly in biopsy specimens. There are a number of IHC markers that can aid in these challenging situations, including AFP, GPC-3, Arg-1, HepPar-1, cpCEA, CD10, BSEP, CD34, HSP70, and Alb-ISH (albumin RNA ISH). However, these markers possess major limitations in that they are markers of hepatic differentiation (Hepar-1, Arg-1, Alb-ISH, pCEA, CD10, and BSEP) or markers of malignancy (GPC-3, CD34, AFP, HSP70).^22,23^ Markers that serve both purposes and can identify malignant hepatocytes are few (AFP and GPC-3), but both exhibit low sensitivity and specificity. Thus, pathologists in practice often use multiple IHC markers in a given case to resolve these diagnostic issues. Our results demonstrate that AFP mRNA detection by RNAscope is a highly specific marker of both hepatocytic differentiation and malignancy in hepatocytic lesions compared to AFP IHC, GPC3, HepPar-1, and Arg-1. The sensitivity and specificity can be further increased by combining AFP RNAscope with either GPC3 or HepPar-1/Arg-1 IHC in specific cases.

GPC3 is the most commonly used IHC marker to distinguish well-differentiated HCCs from other benign liver tumours.^24,25^ Our results indicate that both AFP RNAscope and GPC3 performed well when distinguishing HCC from FNH and LA, as they both were negative in FNH and LA. AFP RNAscope used in combination with GPC3 can diagnose more HCC cases. Differentiating DN from HCC is always difficult in pathologic diagnosis. Previous studies have reported that GPC3 alone cannot distinguish HCC from DN, and a combination of GPC3, HSP70, and GS has been used for the diagnosis of HCC, where the positive expression of more than two of these three markers suggested a diagnosis of HCC.^26^ Our results demonstrated that AFP mRNA has a specificity of close to 100% in both surgical and biopsy samples. Our data demonstrated AFP mRNA is negative in DN, and a positive expression of RNAscope strongly suggests the diagnosis of HCC.

The clinical significance of differentiation between HCC, ICC, and metastatic cancers cannot be overemphasised. The hepatocytic markers used in clinical practice include IHC for HepPar-1, Arg-1, GPC3, and AFP, and the positive rates for these proteins in HCC are reported at 70–85, 45–95, 60–90, and 30%, respectively.^27,28^ Although AFP RNAscope when used purely as a marker of hepatocytic differentiation is much less sensitive compared to HepPar-1 and Arg-1, its 100% specificity indicates this method has a significant advantage over other markers. Positivity for GPC3, HepPar-1, and Arg-1 in non-hepatocytic malignancies has been well documented throughout the literature.^28–31^ Our results are similar to those of previously published studies, where the positive expression of GPC3 was observed in ICC (3.1%, 1/32) and metastatic cancers (lung adenocarcinoma, squamous cell carcinoma, neuroendocrine tumours) (18.0%, 11/61), and the positive expression of HepPar-1 was observed in 6.25% of ICC (2/32) and 0.19% of lung adenocarcinomas (1/52). In contrast, AFP mRNA was only expressed in HCCs. The sensitivity and specificity in distinguishing HCC from non-hepatocytic lesions could be further improved by the combination of GPC3, HepPar-1, and Arg-1 IHC with AFP RNAscope.

There are many causes of HCC, and these include alcohol, fatty liver disease, aflatoxin, and infection with hepatitis B virus (HBV) or hepatitis C virus. The cases in our study were obtained from three hospitals in China, and the majority of patients with HCC possess HBV infection with LC and exhibit clinical features of elevated serum levels of AFP.^32^ This is far different from the pattern in the USA, Europe, and Japan. Our data also suggest a correlation between AFP mRNA levels and HBV infection (*p* = 0.030). Thus, the diagnostic value of AFP in situ detection by RNAscope in western countries requires further confirmation.

Our results indicated that AFP RNAscope is highly specific and possesses moderate sensitivity. Although the expression level of AFP RNAscope is low in some cases, many studies have demonstrated that RNAscope is a highly specific technology and indicate that expression of 1–3 dots per cell in RNAscope test should be evaluated as positive.^17,21^ Our experience suggests that a well-trained pathologist can provide a good interpretation. In clinical applications, we should look carefully for any positive dots to avoid false negative diagnosis.

In summary, our findings suggest that AFP mRNA detected by RNAscope provides a novel diagnostic biomarker for HCC. In practice, AFP RNAscope can be used as the initial marker in the pathologic workup when assessing hepatocytic mass lesions, and when this marker is positive in an appropriate histologic context, it can allow for a diagnosis of HCC. AFP RNAscope can combine with GPC-3 IHC to differentiate HCC from benign lesions or combine with GPC-3, HepPar-1, and Arg-1 to differentiate HCC from other malignancies. The use of AFP RNAscope to diagnose HCC in biopsy specimens is not only cost-effective but also conserves tissue for further molecular studies in identifying specific targets for personalised treatment.

## Supplementary information

Supplementary Materials

## Data Availability

All data included in this study are available upon request, sent to the corresponding author.
