# Supplementary material for: α-Fetoprotein mRNA in situ hybridisation is a highly specific marker of hepatocellular carcinoma: a multi-centre study
Source: Br J Cancer. 2021 Apr 6;124(12):1988–96. doi: 10.1038/s41416-021-01363-4 (PMC8184895; doi:10.1038/s41416-021-01363-4)
Supplement: Supplementary file 1 — Supplementary Materials [file 41416_2021_1363_MOESM1_ESM.docx]

**Supplementary Materials**

**
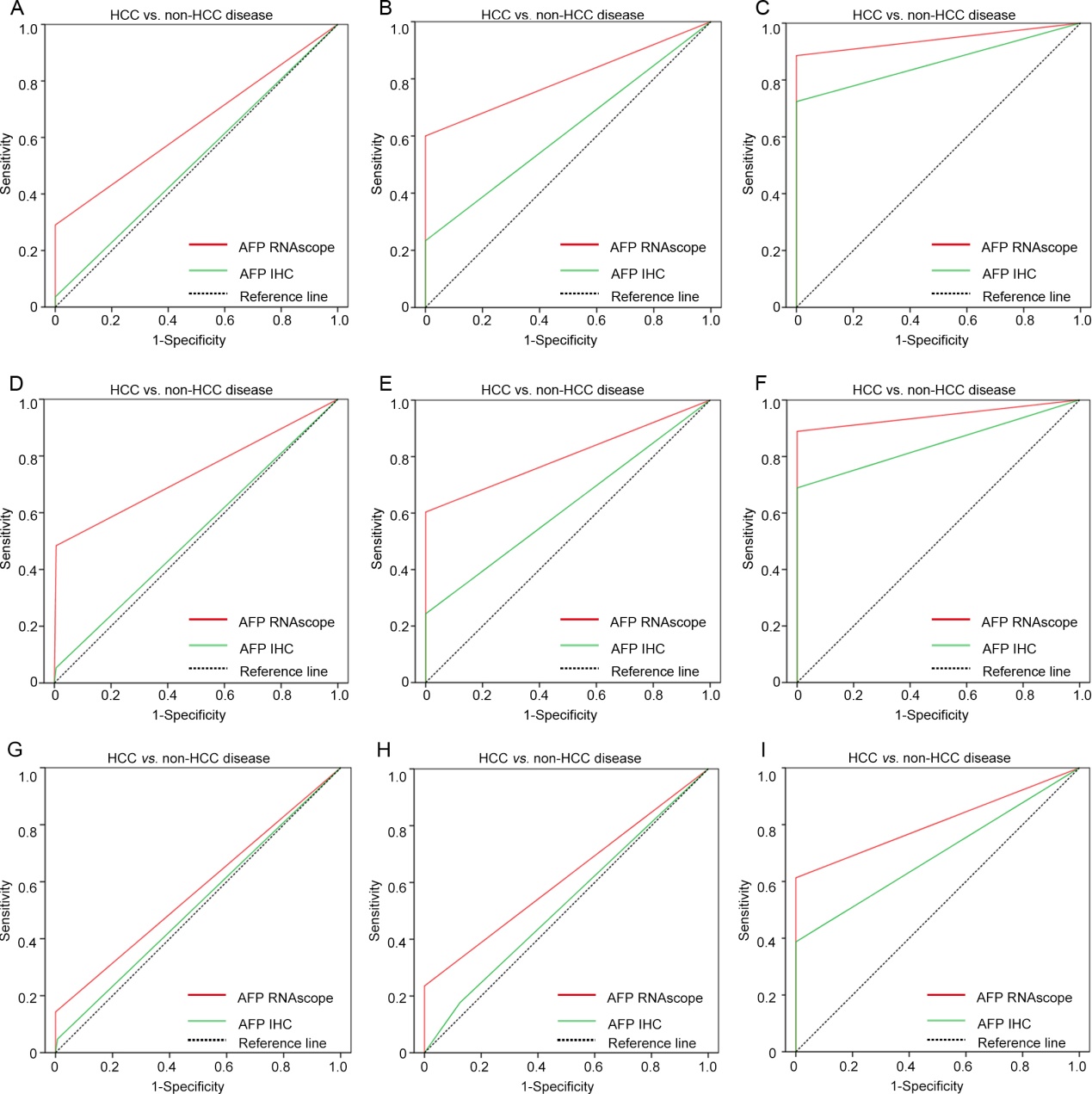
**

**Supplementary Fig. 1. Diagnostic performance of AFP mRNA *in situ* detection in the diagnosis of HCC in different AFP serum level.** ROC curve analyses of AFP RNAscope and AFP IHC in the diagnosis of HCC were shown: AFP ELISA ≤ 20 ng/mL**(A)**, 20 ng/mL ＜ AFP ELISA ≤ 400 ng/mL **(B)** and AFP ELISA > 400 ng/mL **(C)** in the training cohort. AFP ELISA ≤ 20 ng/mL **(D)**, 20 ng/mL ＜ AFP ELISA ≤ 400 ng/mL **(E)** and AFP ELISA > 400 ng/mL **(F)** in the validation cohort. AFP ELISA ≤ 20 ng/mL **(G)**, 20 ng/mL ＜ AFP ELISA ≤ 400 ng/mL **(H)** and AFP ELISA > 400 ng/mL **(I)** in the test cohort.

**
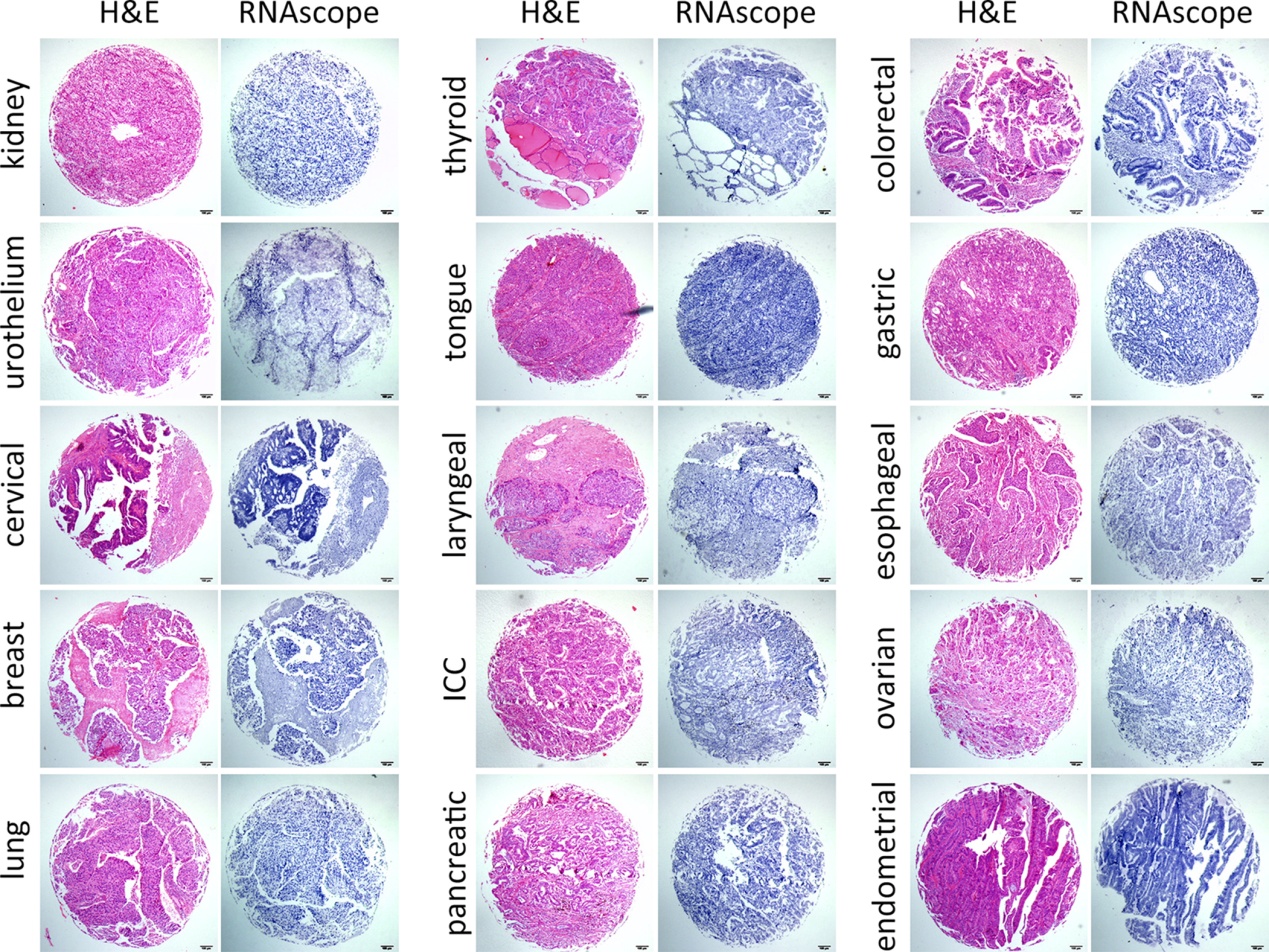
Supplementary Fig. 2. The expression of AFP RNAscope was negative in 15 types of non-hepatoctytic malignancy.** A total of 392 cases, including kidney (n=9), urothelium (n=9), cervical (n=8), breast (n=16), lung (n=15), thyroid (n=12), tongue (n=9), laryngeal (n=9), pancreatic (n=9), colorectal (n=18), gastric (n=16), esophageal (n=13), ovarian (n=11), endometrial (n=9) cancers and intrahepatic cholangiocarcinoma (ICC) (n=229) were used. Representative images for H&E staining and RNAscope were presented.

**Supplementary Table 1.** Results for measurement of AFP by RNAscope, IHC in the diagnosis of HCC in different serum AFP level .

|  | **Training cohort** | | | **Validation cohort** | | | | **Testing cohort** | | |
| --- | --- | --- | --- | --- | --- | --- | --- | --- | --- | --- |
|  | AUC（95%CI） | Sensitivity | Specificity | AUC（95%CI） | Sensitivity | | Specificity | AUC（95%CI） | Sensitivity | Specificity |
| **Cases with serum AFP ≤ 20 ng/mL** | | | | | | | |  |  |  |
| AFP RNAscope | 0.645（0.602-0.688） | 0.290 | 1.000 | 0.739（0.686-0.791） | | 0.484 | 0.994 | 0.571（0.429-0.714） | 0.143 | 1.000 |
| AFP IHC | 0.518（0.475-0.561） | 0.036 | 1.000 | 0.524（0.463-0.585） | | 0.054 | 0.994 | 0.520（0.384-0.656） | 0.048 | 0.993 |
| **Cases with serum AFP 20 - 400 ng/mL** | | | | | | | | | | |
| AFP RNAscope | 0.800（0.733-0.867） | 0.600 | 1.000 | 0.802（0.674-0.929） |  | 0.604 | 1.000 | 0.618（0.394-0.842） | 0.235 | 1.000 |
| AFP IHC | 0.617（0.507-0.726） | 0.233 | 1.000 | 0.622（0.397-0.846） |  | 0.243 | 1.000 | 0.526（0.282-0.774） | 0.176 | 0.875 |
| **Cases with serum AFP ≥ 400 ng/mL** | | | | | | | | | | |
| AFP RNAscope | 0.943（0.907-0.979） | 0.886 | 1.000 | 0.944（0.873-1.000） | | 0.889 | 1.000 | 0.806（0.630-0.983） | 0.613 | 1.000 |
| AFP IHC | 0.862（0.785-0.939） | 0.724 | 1.000 | 0.844（0.661-1.000） | | 0.689 | 1.000 | 0.694（0.41-0.957） | 0.387 | 1.000 |

AUC, area under curve; 95%CI, 95% confident interval; AFP, alpha-fetoprotein; IHC, immunohistochemistry.
